# Mechanical Properties of Nano-TiO_2_-Modified Concrete Under Freeze–Thaw Environment

**DOI:** 10.3390/nano15161254

**Published:** 2025-08-14

**Authors:** Chao Xu, Lin Deng, Dingtao Yang

**Affiliations:** 1 School of Architecture and Civil Engineering, Huangshan University, 39 Xihai Road, Huangshan 245041, China; 2 National and Local Joint Engineering Laboratory, 292 Ziyun Road, Hefei 230009, China; 3 Department of Engineering Geology, Technical University of Berlin, Ernst-Reuter-Platz 1, 10587 Berlin, Germany; dingtao.yang@campus.tu-berlin.de

**Keywords:** nano, freeze–thaw cycle, compressive strength, PFC^2D^

## Abstract

This study investigated the freeze–thaw resistance of ordinary and nano-TiO_2_-modified concrete (NTC) based on mass loss, ultrasonic velocity, compressive strength, and fracture toughness. The compressive behavior and internal damage evolution were further analyzed using particle flow code in two dimensions (PFC^2D^) simulations. The results show that, although neither material exhibited structural collapse after freeze–thaw cycling, visible surface damage was observed, particularly in ordinary concrete. After 100 cycles, NTC showed a 52.17% reduction in mass loss and a 37.31% increase in ultrasonic velocity compared to ordinary concrete. Compressive strength of ordinary concrete decreased by 24.28 MPa (from 41.53 MPa to 17.25 MPa), while that of NTC decreased by only 13.37 MPa, demonstrating that the incorporation of nano-TiO_2_ effectively improves the compressive performance of concrete under freeze–thaw conditions. Fracture toughness after 100 cycles decreased by 89.7% in ordinary concrete and 80.9% in NTC, suggesting that while nano-TiO_2_ mitigates damage, its effect on maintaining fracture load-carrying capacity remains limited. The PFC^2D^ simulations were consistent with the experimental results, effectively capturing peak compressive behavior and validating the model’s applicability for freeze–thaw degradation analysis.

## 1. Introduction

In recent years, the rapid growth of infrastructure construction in cold and wet regions has placed increasingly stringent demands on the durability of concrete materials. Under such climatic conditions, concrete structures are frequently subjected to freeze–thaw cycles, during which the freezing of pore water within the cement matrix induces internal microcracking, surface scaling, and progressive deterioration of mechanical properties [1,2,3,4]. Repeated freeze–thaw action can significantly shorten the service life of concrete structures, making improvement of freeze–thaw resistance a pressing concern in material design and engineering applications.

Traditional approaches to enhancing freeze–thaw durability include air-entrainment, reduced water–cement ratios, and the use of supplementary cementitious materials such as fly ash and slag [5,6,7,8]. These strategies aim to refine the pore structure and reduce permeability, thereby limiting moisture ingress and internal ice expansion. In recent years, nanomaterials have received growing attention due to their superior size effect and potential to modify the microstructure of cementitious composites. Among these nanomaterials, nano-TiO_2_ has been reported to enhance concrete durability by filling microvoids, refining pore size distribution, and reducing capillary porosity [9]. Studies have shown that nano-TiO_2_ can improve resistance to sulfate attack, carbonation, and drying shrinkage [10]; however, its role in resisting freeze–thaw damage, particularly in terms of fracture behavior and the evolution of internal damage, remains insufficiently explored. While research on titanates has shown promising functional enhancements in other material systems—such as lead zirconate titanate thick films with improved electrical properties for high-frequency transducers [11], photo-responsive vanadium dioxide films with high-spatial-resolution holographic recording capability [12], and aluminum titanate films with excellent corrosion resistance prepared via sol–gel methods [13,14], these advances have not yet been fully translated into the context of nano-titanium dioxide in cementitious materials. This knowledge gap limits our understanding of how such nanomaterials may synergistically enhance freeze–thaw resistance, especially from a fracture mechanics perspective.

To date, most studies on the freeze–thaw performance of nano-modified concrete have focused on macroscopic performance, while limited work has been conducted on micromechanical modeling to clarify the internal damage mechanisms. PFC is more suitable for capturing the complex nonlinear deformation and crack propagation behavior of discontinuous media [15,16]. It can effectively simulate the accumulation of microscopic damage and crack evolution under freeze–thaw cycling, offering unique advantages in modeling failure processes in quasi-brittle materials such as concrete [17].

In this study, a combined experimental and numerical approach is adopted to investigate the freeze–thaw resistance of NTC compared to ordinary concrete. The test program includes measurements of mass loss rate, ultrasonic pulse velocity, compressive strength, and fracture toughness after various freeze–thaw cycles. Furthermore, a PFC^2D^ model incorporating a parallel-bond-contact softening mechanism is used to simulate the compressive behavior of damaged specimens.

## 2. Materials and Methods

### 2.1. Materials

**Figure 1:**
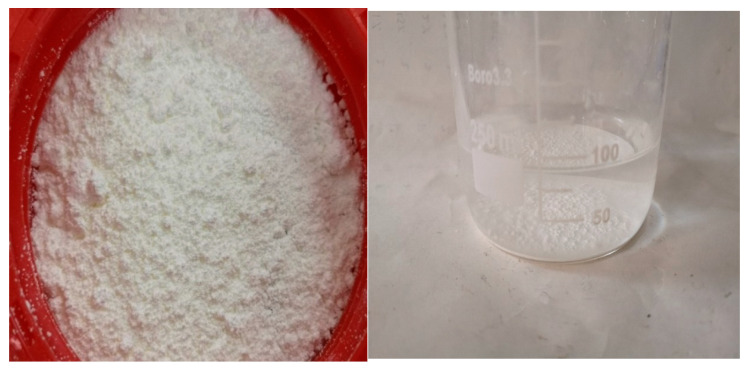
Nano-titanium dioxide and nano-titanium dioxide solution.

The raw materials used in this study included gravel, cement, sand, nano-TiO_2_, a water-reducing agent, and water. The cement employed was a Conch brand ordinary Portland cement with a grade of PO42.5 and a relative density of 3.12 g/cm^3^. The main chemical composition of the cement is presented in Table 1. Natural river sand with a nominal aperture size of 0.6 mm was selected as the fine aggregate. The particle size distribution of the sand was determined using a laser particle size analyzer. The maximum particle size was less than 1.2 mm, with over 99% of particles smaller than 900 μm and more than 50% smaller than 500 μm. The average particle size was 0.46 mm, ensuring good gradation for use in cementitious composites. Coarse aggregate was prepared using continuously graded crushed limestone with particle sizes ranging from 5 to 20 mm. The crushed stone had a surface density of 2700 kg/m^3^ and a crushing index of 4.8%, indicating favorable mechanical strength and durability. The titanium dioxide was white, as shown in Figure 1; the details of nano-TiO_2_ are shown in Table 2.

### 2.2. Specimen Preparation

To begin with, preliminary exploratory tests were conducted, which revealed that nano-TiO_2_ exhibits strong water absorption. When the nano-TiO_2_ content reached 5% by mass of cement, the concrete mixture became difficult to stir and shape due to poor workability. Therefore, the incorporation of a water-reducing agent was deemed necessary to ensure proper mixing and molding of the NTC. To minimize the influence of the water reducer on the experimental results, both experimental groups were controlled to use the same amount (4.1 g) of water-reducing agent. In this study, the content of nano-TiO_2_ was fixed at 3% of the cement mass, as 3% achieved the best balance of flowability and mechanical performance in the preliminary tests. Based on the mix design in Table 3, the nano-TiO_2_, the water reducer, and a portion of the mixing water were first weighed and pre-mixed to prepare a nano-TiO_2_ dispersion solution. This step was crucial to achieving a uniform distribution of nanoparticles within the concrete matrix, as nanoparticle agglomeration or sedimentation can significantly affect the performance of the final composite. The gravel, cement, and sand were then weighed and dry-mixed in a 5 L cement mortar mixer at low speed for 2–4 min to ensure homogeneous blending. After the dry mixing was complete, the pre-prepared nano-TiO_2_ solution was added to the mixture, followed by the gradual addition of the remaining two-thirds of the mixing water. The entire mixture was then stirred at high speed for an additional 3–5 min. The mixing process was considered complete when the mixture achieved suitable workability, characterized by a uniform, non-agglomerated paste with adequate flowability. The resulting fresh concrete was then poured into standard molds to form test specimens. Each specimen was cubic in shape, with dimensions of 100 mm × 100 mm × 100 mm, and these specimens were used for subsequent compressive-strength testing.

### 2.3. Freeze–Thaw Test Design and Surface Deterioration Analysis

After specimen preparation, all samples were cured under standard conditions for 28 days, after which their initial mass was recorded. During the freeze–thaw testing, the specimens were placed in a dedicated freeze–thaw chamber, with each container filled with clean water such that the water level remained at least 5 mm above the specimen surface, ensuring full saturation throughout the process. Each freeze–thaw cycle lasted 4 h, and every 25 cycles was defined as a single testing stage. At the end of each stage (i.e., after every 25 cycles), the specimens were removed and surface-dried at room temperature, and their mass was remeasured. The testing then proceeded to the next stage. Mass measurements were taken after 50, 75, and 100 cycles, and the mass loss rate along with the ultrasonic pulse velocity were calculated at each interval to evaluate the freeze–thaw resistance and internal structural integrity of the specimens.

**Figure 2:**
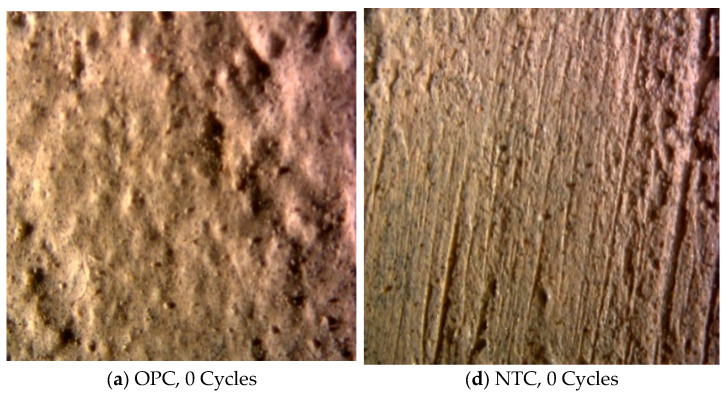

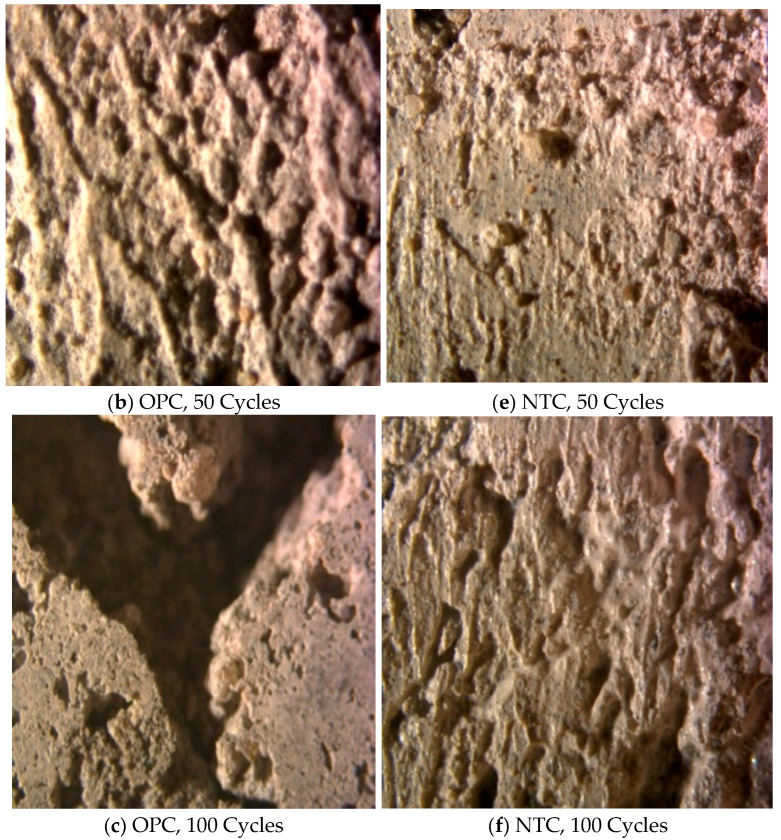
Microscopic surface characteristics of OPC and NTC.

Macroscopically, no significant damage was observed on the specimens following freeze–thaw exposure. However, microstructural deterioration was examined using a stereomicroscope at 60× magnification, as illustrated in Figure 2. Under ambient conditions, the surface of the NTC was notably smoother compared to that of the plain concrete. After 50 freeze–thaw cycles, the plain concrete surface began exhibiting pronounced roughness and irregularity. By the 100th cycle, large surface pores had developed. This progressive deterioration is primarily attributed to the freezing and expansion of water within the capillary pores of the cement matrix at low temperatures. Repeated freeze–thaw cycling led to gradual enlargement and collapse of these pores. Under magnification, the initiation and propagation of surface porosity in the plain concrete were clearly visible.

In contrast, the NTC group displayed minimal surface roughening even after 100 cycles, indicating a significantly lower degree of freeze–thaw degradation. This enhanced performance is mainly ascribed to the incorporation of nano-TiO_2_, which effectively filled larger voids within the cementitious matrix, thereby reducing moisture ingress and subsequent ice-induced expansion. Consequently, the modified concrete exhibited markedly improved resistance to freeze–thaw damage.

## 3. Results and Discussion

### 3.1. Mass Loss Rate

**Figure 3:**
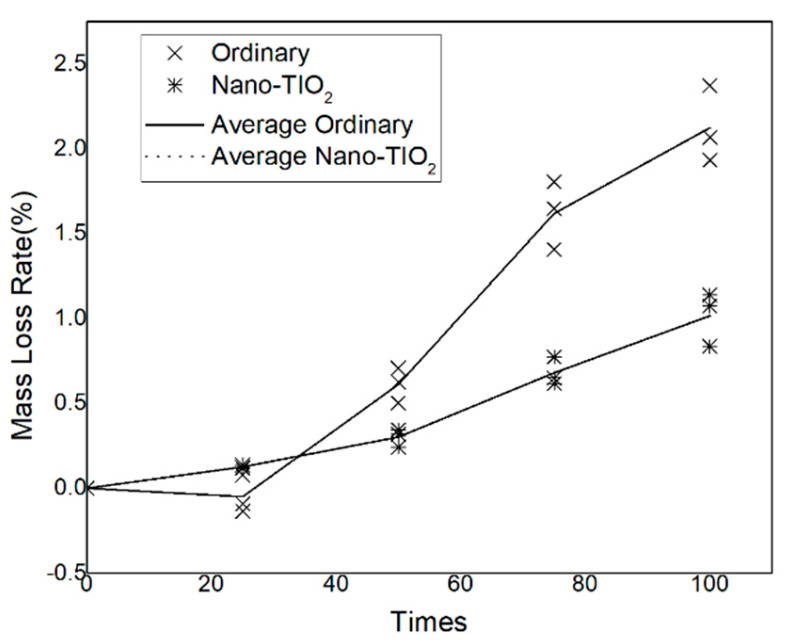
The influence of freeze–thaw cycling on the concrete mass-loss rate.

The mass loss of concrete specimens serves as a direct indicator of changes in volume and density resulting from freeze–thaw (F–T) cycling, and provides a quantitative measure of concrete durability degradation under such environmental stress. The mass loss rates of the specimens subjected to F–T cycles are presented in Figure 3.

For ordinary concrete, the mass loss rate exhibits a non-monotonic trend with the number of F–T cycles: initially decreasing and subsequently increasing. In contrast, the NTC displays a continuous upward trend in mass loss rate throughout the F–T process.

A notable observation is that ordinary concrete exhibits a negative mass loss rate after 25 F–T cycles, indicating a slight increase in specimen mass. This phenomenon is attributed to the expansion of internal pores caused by freezing, which facilitates the ingress of additional water or solution into the concrete matrix, thereby slightly increasing its mass. However, with further F–T cycles, the absorption effect becomes less significant, and surface scaling and material loss become dominant, leading to a progressive increase in mass loss rate. After 100 F–T cycles, the average mass loss rate of ordinary concrete reaches 2.124%, which aligns with prior microscopic evidence of surface porosity and degradation.

In the case of NTC, the mass loss rate after 20 F–T cycles is only 0.126%, indicating excellent durability in the early stages of cycling. As the number of F–T cycles increases, the mass loss rate rises gradually, reaching 1.016% after 100 cycles. Compared to ordinary concrete, this represents a 52.17% reduction in mass loss, underscoring the effectiveness of nano-TiO_2_ incorporation in enhancing resistance to long-term F–T deterioration. These results demonstrate that nano-TiO_2_ significantly improves the integrity and durability of concrete under repeated freeze–thaw exposure by mitigating mass loss.

### 3.2. Ultrasonic Wave Velocity

**Figure 4:**
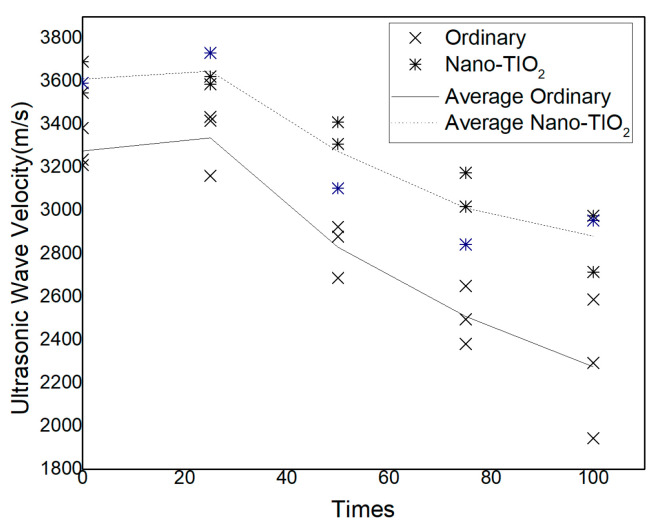
The influence of freeze–thaw cycles on the concrete ultrasonic wave velocity.

Figure 4 illustrates the ultrasonic pulse velocity (UPV) of different concrete types following various numbers of freeze–thaw cycles.

It can be observed that with increasing freeze–thaw cycles, both ordinary concrete and NTC exhibit a similar overall trend of UPV evolution. After 25 freeze–thaw cycles, the UPV of both concretes increases slightly, followed by a gradual decline as the cycles progress. Specifically, the UPV of ordinary concrete reaches its peak value of 3337.33 m/s at 25 cycles, while the UPV of NTC attains a higher peak value of 3646.67 m/s.

As the number of cycles increases, internal microstructural degradation and pore development lead to a reduction in material compactness. After 100 freeze–thaw cycles, the UPV of ordinary concrete decreases from 3276 m/s to 2274 m/s, a total reduction of 1001 m/s. In comparison, NTC experiences a smaller decline, from 3610 m/s to 2881 m/s, corresponding to a reduction of 729 m/s. Notably, the UPV of NTC remains 37.31% higher than that of ordinary concrete after 100 cycles, indicating that the incorporation of nano-TiO_2_ significantly enhances the structural integrity and compactness of concrete under long-term freeze–thaw action.

Additionally, it is worth noting that after 100 freeze–thaw cycles, the UPV values of ordinary concrete exhibit increased variability. This is primarily attributed to surface irregularities caused by progressive freeze–thaw damage, which adversely affect the accuracy and consistency of ultrasonic measurements. Although the specimen surfaces were polished using sandpaper to reduce measurement errors, the dispersion remained relatively high.

### 3.3. Three-Point Bending Test

**Figure 5:**
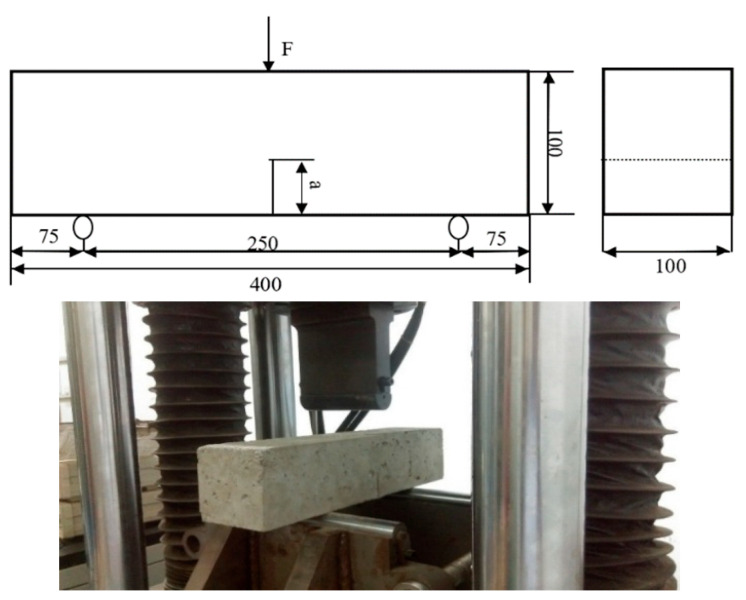
Specimen design dimensions for three-point flexural test.

The experimental design adopted a three-point bending beam specimen with a pre-fabricated central notch and a single concentrated load applied at the mid-span. The specimen dimensions were 400 mm × 100 mm × 100 mm, as shown in Figure 5. A single notch of length a was introduced at the mid-span to serve as the initial crack.

Preliminary testing indicated that when the notch length a was 50 mm, some specimens experienced complete fracture after freeze–thaw exposure, rendering them unsuitable for subsequent three-point bending tests. Therefore, to ensure test continuity while maintaining crack sensitivity, a notch length of 40 mm was selected for all formal experiments. The actual specimen configuration is illustrated in Figure 4 [18].

This design enables the controlled study of crack propagation and fracture behavior in concrete subjected to freeze–thaw cycles while ensuring structural integrity during mechanical loading.

According to the formula recommended by ASTM standards, the fracture toughness $K_{IC}$ of concrete can be calculated based on the experimentally obtained peak load $F_{max}$. For single-edge notched-beam specimens subjected to three-point bending, the fracture toughness is determined using the following expression:

(1) $$K_{IC}=\frac{P_{max}S}{bh^{\frac{3}{2}}}f(\frac{a_{0}}{h})$$

(2) $$f\left(\frac{a_{0}}{h}\right)=2.9{\left(\frac{a_{0}}{h}\right)}^{\frac{1}{2}}-4.6{\left(\frac{a_{0}}{h}\right)}^{\frac{3}{2}}+21.8{\left(\frac{a_{0}}{h}\right)}^{\frac{5}{2}}-37.6{\left(\frac{a_{0}}{h}\right)}^{\frac{7}{2}}+38.7{\left(\frac{a_{0}}{h}\right)}^{\frac{9}{2}}$$

where 

$a_{0}$: initial crack length (mm);

S: specimen span (mm);

b: specimen width (mm);

h: specimen height (mm).

The fracture toughness of ordinary concrete and NTC after different numbers of freeze–thaw cycles can thus be determined, as summarized in Table 4.

Without undergoing freeze–thaw cycles, the average fracture toughness of ordinary concrete was measured to be 1.58 MPa·m^1/2^, while that of NTC reached 1.84 MPa·m^1/2^, indicating a slight enhancement in fracture resistance under normal conditions due to the incorporation of nano-TiO_2_.

After 100 freeze–thaw cycles, the fracture toughness of ordinary concrete dropped significantly. For example, specimen 1 exhibited a fracture toughness of 0.11 MPa·m^1/2^, while specimen 3 reached 0.35 MPa·m^1/2^, showing considerable variation among replicates. On average, the maximum fracture load-carrying capacity of ordinary concrete decreased by approximately 89.7% compared to concrete in the unfrozen state.

For NTC, the average fracture toughness after 100 cycles was 0.26 MPa·m^1/2^, corresponding to a relative reduction of 80.9%. Although nano-TiO_2_ helps mitigate freeze–thaw damage to some extent, its effect on preserving fracture load-carrying capacity remains limited.

These results clearly demonstrate that freeze–thaw cycling has a substantial impact on the fracture resistance of concrete, significantly reducing its crack propagation threshold. While nano-TiO_2_ slightly improves the initial fracture toughness, it does not fundamentally alter the fracture mode governed by crack opening. which is the primary factor controlling the maximum fracture load of concrete.

### 3.4. Compressive Strength

**Figure 6:**
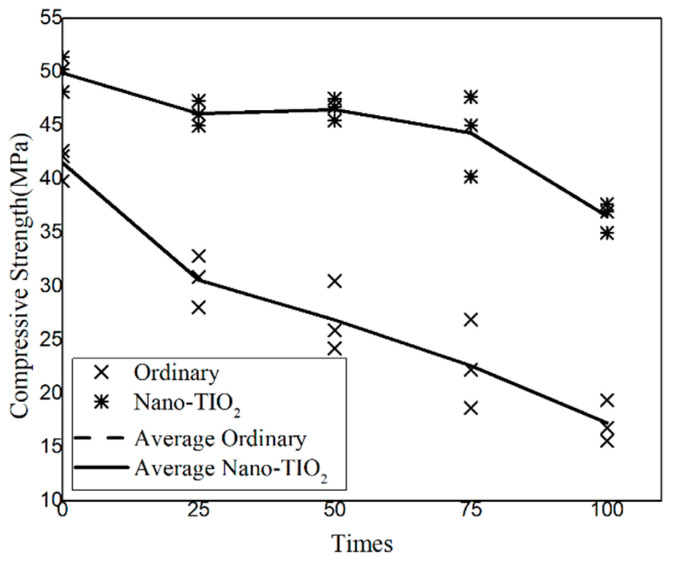
The influence of dry–wet cycles on concrete’s compressive strength.

Figure 6 presents the compressive strength of different concretes after freeze–thaw cycles. As shown, the average peak compressive strength of both ordinary concrete and NTC exhibits a decreasing trend with increasing freeze–thaw cycles. Initially, the ordinary concrete demonstrated an average maximum compressive strength of 41.53 MPa, while the NTC group achieved 49.91 MPa—indicating a 20.17% enhancement due to the incorporation of nano-TiO_2_. After 100 freeze–thaw cycles, the compressive strength of the ordinary concrete decreased from 41.53 MPa to 17.25 MPa, a reduction of 24.28 MPa, with the most pronounced decline occurring within the first 25 cycles. In contrast, the compressive strength of the NTC specimens dropped from 49.91 MPa to 36.54 MPa, a reduction of 13.37 MPa, with the most significant decrease observed between 75 and 100 cycles. After 100 cycles, the reduction in compressive strength of the NTC specimens was 44.93% less than that of the ordinary concrete, highlighting their improved freeze–thaw resistance.

**Figure 7:**
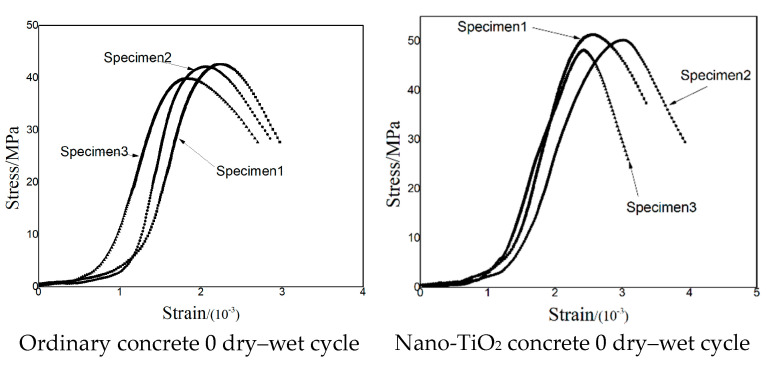

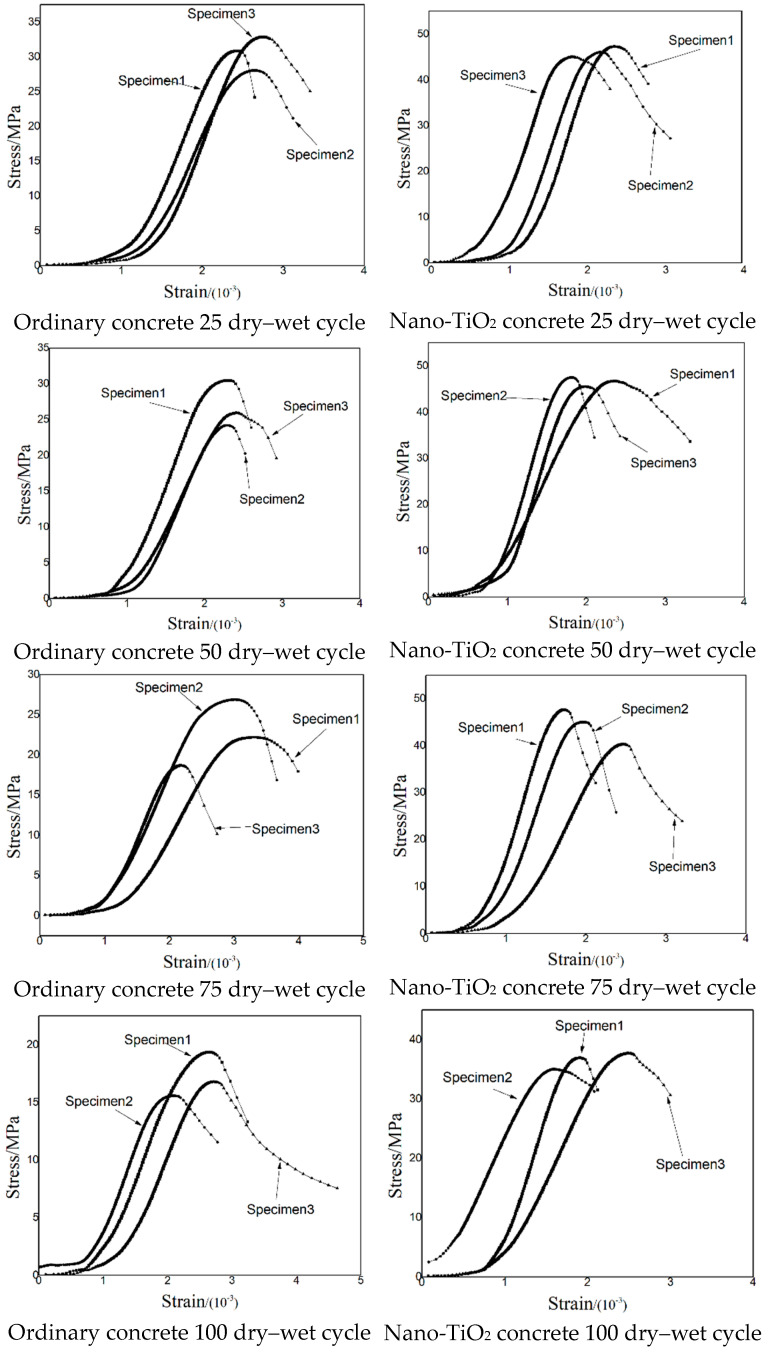
The compressive stress–strain curve of the specimen was obtained through uniaxial compression tests.

Figure 7 illustrates the compressive stress–strain curves of ordinary concrete and NTC specimens after freeze–thaw cycles. It can be observed that freeze–thaw cycling has a relatively minor impact on the overall stress–strain behavior of the NTC-modified concrete. The stress–strain curve of NTC exhibits the typical three-stage behavior found in ordinary concrete: (1) compaction stage, (2) elastic stage, and (3) yield stage. However, compared with ordinary concrete, NTC specimens show reduced dispersion in stress–strain responses under the same number of freeze–thaw cycles. This indicates that the incorporation of nano-TiO_2_ improves the compressive stability of concrete subjected to repeated freeze–thaw action. With increasing freeze–thaw cycles, the peak strain of ordinary concrete gradually decreased, indicating a loss in ductility. In contrast, the NTC specimens exhibited a slight increase in peak strain compared to the initial value. The freeze–thaw durability of cementitious materials largely depends on their transport properties, such as permeability and diffusivity, which are governed by the total pore volume, pore size distribution, shape, and connectivity. The addition of nano-TiO_2_ effectively reduces the porosity and permeability of the concrete matrix, thereby limiting water ingress and associated freeze-induced stress damage. As a result, the material experiences less structural degradation and demonstrates a modest improvement in ductility, confirming that nano-TiO_2_ enhances the freeze–thaw resistance of concrete.

## 4. Compression Simulation

### 4.1. Generation and Servo of Compression Specimens

**Figure 8:**
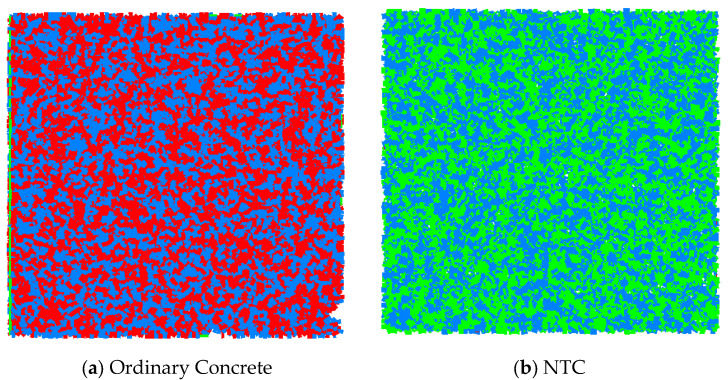
Simulation contact of specimens.

Numerical simulations were performed using PFC^2D^, a discrete element method software. In the modeling process, coarse aggregate particles were represented as spherical elements. The process includes defining the model domain, walls, particles, and initial properties, followed by iterative equilibrium and model testing. The parameters are calibrated by comparing the simulation results with the experimental data, including multiple loading tests to ensure model response accuracy. The specimen model had dimensions of 100 mm × 100 mm. For the ordinary concrete specimen, a total of 7080 particles were generated. As shown in Figure 8, which presents the contact network for both ordinary concrete and NTC specimens, the ordinary concrete model formed 14,169 contacts, whereas the NTC model generated 20,316 contacts. This increase in contact number reflects the higher particle density and improved packing achieved through the incorporation of nano-TiO_2_, which contributes to enhanced matrix compactness and mechanical stability in the modified concrete.

Interparticle interaction was modeled using the parallel-bond model. In this framework, contact forces arise from the overlapping of adjacent particles, while the parallel bonds provide additional normal and shear stiffness as well as moment transfer capability. When bonded, the contact behaves in a linearly elastic manner and can resist torque, until the applied stress or moment exceeds the bond strength threshold, at which point the bond is considered broken, simulating the formation of microcracks.

During the initialization stage, the contact configuration among particles lacked sufficient precision, resulting in a mechanically unstable system due to excessive interparticle interaction. To resolve this, a servo-control mechanism was implemented to balance internal forces iteratively. When the ratio of the average unbalanced force to the average contact force dropped below 1 × 10^−5^, the system was deemed to have reached quasi-static equilibrium, and the model was considered ready for subsequent mechanical simulations.

The micromechanical parameters used for both concrete types are listed in Table 5, along with the established correspondence between macroscopic mechanical behavior (e.g., elastic modulus, tensile strength) and microscopic bond properties. In the modeling process, the influence of freeze–thaw damage was simulated by differentiating between intact and degraded bonds. Specifically, two parallel-bond parameter sets were used to reflect material softening due to freeze–thaw cycles.

For ordinary concrete, the undamaged parallel-bond parameters were set as follows: effective modulus: 28.05 GPa; tensile strength: 25 MPa; bond strength: 100 MPa. After 100 freeze–thaw cycles, 50% of the particle bonds were assigned degraded parameters: effective modulus: 10 GPa; tensile strength: 5 MPa; bond strength: 50 MPa. For the NTC specimens, the initial (intact) bond parameters were as follows: effective modulus: 28.05 GPa; tensile strength: 31 MPa; bond strength: 100 MPa. After 100 cycles, the degraded 50% of the bonds were set as follows: effective modulus: 10 GPa; tensile strength: 20 MPa; bond strength: 90 MPa. These values reflect the enhanced freeze–thaw resistance and reduced microstructural degradation of the NTC. By establishing a mapping between macro-scale mechanical performance and mesoscopic simulation parameters, the model can effectively capture the degradation mechanisms and durability differences between ordinary and nano-modified concretes under freeze–thaw action.

### 4.2. Analysis of the Simulation Results

**Figure 9:**
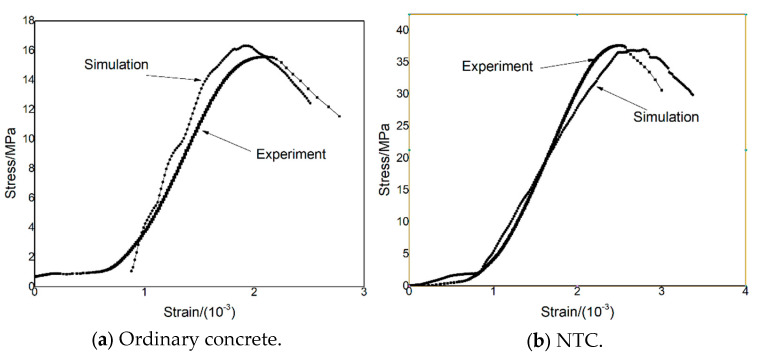
Test and simulated stress–strain diagrams of specimens.

Figure 9 presents a comparison between experimental and simulated stress–strain curves for the two types of concrete. While some differences are observed in the overall curve trends, this deviation is primarily attributed to the inherent variability and scatter in the stress–strain behavior of concrete specimens during physical testing. Therefore, to evaluate the accuracy and reliability of the numerical model, the comparison focuses on the peak compressive stress and the corresponding peak strain values.

After 100 freeze–thaw cycles, the experimental peak compressive strength of ordinary concrete was measured at 15.59 MPa, with a corresponding peak strain of 2.08 × 10^−3^. The simulated results yielded a peak compressive strength of 16.30 MPa and a peak strain of 1.96 × 10^−3^, resulting in a 4.55% deviation in peak stress and a 5.77% deviation in peak strain between the simulation and experiment.

For the NTC, the experimental peak compressive strength after 100 cycles was 37.67 MPa, with a peak strain of 2.50 × 10^−3^. The corresponding simulation results produced a peak stress of 37.01 MPa and a peak strain of 2.78 × 10^−3^, corresponding to deviations of 1.75% in peak stress and 11.20% in peak strain.

These results demonstrate that the numerical model can effectively reproduce the mechanical behavior of the tested materials, particularly with respect to capturing the peak compressive strength and the associated strain levels. The close agreement between the simulated and experimental peak values confirms the validity and applicability of the model in evaluating the mechanical degradation of concrete subjected to freeze–thaw cycles, and highlights the superior freeze–thaw resistance of NTC.

**Figure 10:**
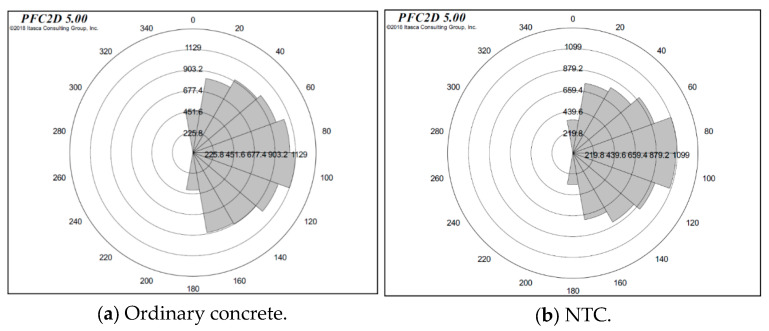
Angle of microcracks in ordinary concrete and NTC under simulated compression test after 100 freeze–thaw cycles.

Figure 10 illustrates the statistical distribution of microcrack orientation and quantity in simulated specimens of ordinary concrete and NTC after undergoing freeze–thaw cycles. The results reveal that the ordinary concrete specimen developed a total of 8587 cracks, while the NTC specimen exhibited 7788 cracks, indicating a higher crack density and internal damage in the ordinary concrete.

In the ordinary concrete, most cracks were oriented between 20° and 160°, with a concentration at 90°, where the number of cracks reached 1129, suggesting that vertical cracking was the predominant mode under uniaxial compressive loading. The crack lengths ranged from 0.09 to 0.15 (normalized or in millimeters, depending on context). Even with bond softening in part of the particles due to freeze–thaw degradation, vertical cracks remained dominant. However, a notable increase in inclined crack distribution was observed, reflecting more complex fracture mechanisms induced by internal damage.

Furthermore, the simulation results show that bond degradation led to a significant increase in the total number of microcracks during failure, indicating that freeze–thaw cycling exacerbates crack initiation and propagation. This underscores the importance of mitigating microcrack formation in concrete structures exposed to cyclic freezing and thawing, as these cracks can coalesce and evolve into macroscopic fractures, compromising structural integrity over time.

For the NTC-modified concrete, cracks were also primarily distributed between 20° and 160°, with the highest count at 90°, totaling 1099 cracks. The crack lengths ranged from 0.02 to 0.149, and were generally shorter than those observed in ordinary concrete. Although the addition of nano-TiO_2_ did not alter the principal orientation of crack propagation under compressive loading, it significantly reduced the average crack length, thereby limiting the development of microcracks and suppressing their progression into macrocracks. This enhancement in microstructural integrity effectively contributes to the improved freeze–thaw resistance of NTC.

In summary, while the primary fracture orientation remained similar, the NTC exhibited fewer and shorter cracks, indicating its superior durability. These findings provide valuable insights for the design of durable concrete materials in cold-region engineering applications, where resistance to freeze–thaw-induced cracking is critical.

## 5. Conclusions

In this study, the mass loss rate and ultrasonic pulse velocity of ordinary concrete and NTC-modified concrete were measured after 25, 50, 75, and 100 freeze–thaw cycles. Mechanical performance was evaluated through uniaxial compression tests and three-point bending tests with pre-cracked beams. The compressive behavior of freeze–thaw-damaged specimens was also simulated using a PFC^2D^ particle contact softening model.

Although the overall structure remained intact after cycling, surface deterioration was evident. The addition of nano-TiO_2_ effectively reduced such damage. After 100 cycles, NTC showed 52.17% less mass loss and a 37.31% increase in ultrasonic velocity compared to ordinary concrete, indicating better structural integrity. For fracture behavior, the maximum fracture load of ordinary concrete decreased by 89.7% after 100 cycles. NTC showed an 80.9% reduction in fracture toughness, dropping to 0.26 MPa·m MPa·m^1/2^. Although nano-TiO_2_ improved crack resistance to some extent, its ability to maintain fracture capacity under severe conditions was limited. The compressive strength of ordinary concrete dropped from 41.53 MPa to 17.25 MPa (−24.28 MPa), while NTC decreased from 49.91 MPa to 36.54 MPa (−13.37 MPa), showing improved freeze–thaw resistance. PFC^2D^ simulation effectively reproduced the material’s mechanical behavior, particularly in capturing its peak compressive strength, confirming the model’s reliability.

## Figures and Tables

**Table 1:** Chemical composition of cement.

Component	SiO_2_	Al_2_O_3_	CaO	Fe_2_O_3_	MgO	SO_3_
Cement	22.02	5.2	64.42	5.23	1.02	2.1

**Table 2:** Nano-TiO_2_ properties.

Properties	Density	Melting Point/°C	Boiling Point/°C	Particle Size/nm
Nano-TiO_2_	4.26	1855	2900	25

**Table 3:** Mixture ratio of the nano-TiO_2_-modified concrete (unit: %).

Group	Cement	Sand	Gravel	Water	Water Reducer	Nano-TiO_2_
Ordinary concrete	17.27	24.26	50.93	7.37	0.17	0
Nano-TiO_2_-modified concrete	16.76	24.26	50.93	7.37	0.17	0.51

**Table 4:** Relationship between freeze–thaw cycles and fracture toughness (MPa·m^1/2^).

Type	Specimen No.	0	25	50	75	100
Ordinary Concrete	1	1.63	1.31	0.79	0.46	0.11
	2	1.70	1.39	0.68	0.66	0.17
	3	1.41	1.21	1.05	0.77	0.35
	Average	1.58	1.30	0.84	0.63	0.21
NTC	1	1.86	1.73	0.84	0.51	0.18
	2	2.09	1.40	1.15	0.68	0.39
	3	1.99	1.62	0.97	0.64	0.50
	Average	1.84	1.48	0.94	0.61	0.26

**Table 5:** Simulation parameters of compression for specimen.

Parameter	Ordinary Concrete	NTC
Minimum radius/mm	0.045	0.01
Maximum radius/mm	0.075	0.075
Normal stiffness/N·m^−1^	1 × 10^9^	1 × 10^9^
Shear stiffness//N·m^−1^	1 × 10^9^	1 × 10^9^
Friction factor	0.577	0.577
Parallel effective modulus/GPa	28.05	28.05
Tensile strength/MPa	25	31
Cohesion/MPa	100	100
Linear effective modulus/GPa	99.33	99.33
Parallel effective modulus after freeze–thaw/GPa	10	10
Tensile strength after freeze–thaw/MPa	5	20
Cohesion after freeze–thaw/MPa	50	90

## Data Availability

Data are contained within the article.
